# Electronic cigarettes use and perception amongst medical students: a cross sectional survey from Sindh, Pakistan

**DOI:** 10.1186/s13104-018-3303-z

**Published:** 2018-03-22

**Authors:** Nousheen Iqbal, Zain A. Khan, Syed Muhammad Hamza Anwar, Omar Irfan, Babar Irfan, Ammar Mushtaq, Maria Bibi, Faraz Siddiqui, Javaid A. Khan

**Affiliations:** 1 Section of Pulmonology and Critical Care, Department of Medicine, Aga Khan University Hospital, Karachi, Pakistan; 2 Dow University of Health Science, Karachi, Pakistan; 3 Aga Khan University, Karachi, Pakistan; 4 Jinnah Sindh Medical University, Karachi, Pakistan; 5 Jinnah Medical and Dental Collage, Karachi, Pakistan; 6 Liaquat University of Medical and Health Sciences, Hyderabad, Sindh Pakistan; 7 Department of Medicine, Aga Khan University Hospital-Karachi, Karachi, Pakistan

**Keywords:** Knowledge, Perception, E-cigarette use, Medical student

## Abstract

**Objective:**

The manufacturers of electronic cigarettes (e-cigarettes) are actively marketing their product through electronic and social media. Undergraduate medical students are expected to have better knowledge and awareness as they directly interact with patients in their training, The purpose of this study is therefore, to determine knowledge, use and perception regarding e-cigarettes among medical students from Sindh, Pakistan.

**Results:**

A cross-sectional study was conducted between 1st July and 30th September 2016 at five different medical colleges situated in the second largest province of Sindh, Pakistan. The data was collected through a structured, self-administered questionnaire. Of the 500 students, the mean age was 21.5 ± 1.7 years and 58% were females. Over (65.6%) students were aware of e-cigarettes, 31 (6.2%) reported having used e-cigarettes, of whom 6 (1.2%) self-reported daily use. Users of conventional tobacco products were significantly more likely to have heard of e-cigarettes (87.6% vs 51.6%, p < 0.001) and having used them (13.9% vs 1.3%, p < 0.001). On multivariable logistic regression analysis we found a strong association of e-cigarette use with consumption of conventional cigarettes [OR: 10.6, 95% CI 3.6–30.8, p < 0.001], use of smokeless tobacco products [OR: 7.9, 95% CI 2.7–23.4, p < 0.001] however a weak association was observed for Shisha use [OR: 3.05, 95% CI 0.9–9.6, p = 0.05].

## Introduction

An electronic cigarette (e-cigarette), also known as electronic nicotine delivery systems (ENDS) is a handheld electronic device that was first introduced in the early 2000s [1]. E-cigarettes have a basic design with engineering variations and user modifications which results in difference in nicotine delivery [2]. It is currently actively marketed with healthy claims, as a better alternative to conventional cigarette smoking and as smoking cessation aid, often endorsed by celebrities and doctors [3, 4].

E-cigarettes have generally been found to be common among smokers with high socioeconomic class who have tried to quit in the past year [5]. Published data from center of disease control and prevention (CDC) suggests that most e-cigarette users are either significantly younger individuals, current smokers or people who have recently quit smoking [6]. E-cigarette use has also been found to be higher among school and college students trying it on an experimental basis or as a safe alternative [7, 8]. Brożek et al. surveyed medical students among whom e-cigarette use was 1.3% with majority of users being male [9]. Although it is perceived effective as a harm reduction tool [10], there is still insufficient evidence in support of e-cigarettes’ efficacy in smoking cessation [11]. Alternatively, use of e-cigarettes can also have negative health consequences; in a cross-sectional survey of students from 24 colleges and universities in Texas found e-cigarette use to be associated with depressive symptoms [12]. Until long term observational data of e-cigarette users is available, possibility of adverse health effects cannot be ruled out.

As the new epidemic of e-cigarettes continues to rise globally, there is little or no data available on the knowledge and use of e-cigarettes from developing regions, which are still struggling to contain the use of conventional tobacco products. This highlights a key knowledge gap that still remains to be addressed. In general, medical students are considered to have better levels of knowledge as they are expected to interact with patients in their training years, so it is important to know regarding their perspectives on e-cigarettes. We therefore, aimed to explore the existing levels of knowledge, attitude and perception as well as usage of e-cigarettes among medical students from two of the largest cities of Sindh Province in Pakistan.

## Main text

### Methods

This was a cross-sectional study conducted between 1st July and 30th September 2016 at five different medical colleges situated in two cities of second largest province of Sindh, Pakistan. This included two private institutions (Aga Khan University, Jinnah Medical and Dental College) and three public sector institutions (Jinnah Sindh Medical University, Dow University of health sciences and Liaquat University of Medical and Health Sciences). Our study population comprised of undergraduate medical students. The study was approved by the ethical review board of all the institutes. Selection of institutions participating in our study was based on ethical and administrative approvals from each site. Within each institute, we obtained a random sample of 100 undergraduate medical students to achieve a total sample size of 500 respondents.

We classified cigarette and e-cigarette use as follows: Never smokers were defined as those who reported to never have smoked. Current smokers were individuals who reported to have smoked at least one cigarette in last month. Occasional smokers were defined as individuals who smoked less than once a month. An Ex-Smoker was defined as a person who had given up smoking and had not smoked in last 3 months.

The data was collected through a self-administered, structured questionnaire which was developed in English. Potential interview participants were initially briefed about the topic beforehand, and a written, informed consent was obtained. A brief socio-demographic section in the questionnaire contained participant’s age, gender, name of college and year of study. The subsequent questions gathered detail regarding participants’ self-reported smoking status, concomitant use of other tobacco products like shisha and smokeless tobacco products and whether they heard about e-cigarette. Respondents who had heard about e-cigarettes were then were asked further questions about e-cigarette use and their perceptions related to its safety and harm. Participants took approximately 5–10 min to fill out the questionnaire, which were then returned to the data collection team. All the collected data were de-identified in order to maintain the confidentiality of study participants and the anonymity of their data at all times.

### Statistical analysis

A descriptive analysis was performed to describe the demographic characteristics as well as the knowledge and use of e-cigarettes, as well as to compare between users and non-users of conventional tobacco products, which included cigarettes, smokeless tobacco and shisha. Quantitative data were presented as mean ± SD while categorical data were expressed as absolute and relative frequencies. We also compared the perception of e-cigarettes between tobacco users and non-users. The differences between these groups was assessed using the Chi square test or students’ t-test where appropriate. We further conducted a binary logistic regression analysis to identify the types of tobacco use associated with e-cigarette consumption (yes/no), adjusting for relevant variables in multivariable analysis. A p-value of < 0.05 was considered as statistically significant for all statistical tests performed. The analysis was performed on STATA v12.0

### Results

A total of 500 students were interviewed which included 290 (58%) females and 210 (42%) males. The mean age for the overall sample was 21.6 ± 1.7 years. We observed conventional forms of tobacco to be more common. Overall, 194 (38.8%) respondents were users of conventional tobacco products either in the form of cigarettes, shisha or smokeless tobacco. Among those who reported smoking, 43 (8.6%) labelled themselves as current cigarette smokers, 41 (8.2%), as occasional smokers and 4 (0.8%) were ex-smokers. Respondents who used conventional tobacco products were significantly more likely to be males (63.5% vs 28.4%, p < 0.001), and enrolled in public sector institutes (60.4% vs 39.6%, p < 0.001). More than 50% of these tobacco users belonged to 4th and 5th year of medical school (p < 0.03).

E-cigarette use in our sample was reported by 31 (6.20%) respondents; 27 (87.1%) of these also reported cigarette smoking. It was observed that an overall majority (65.6%) of students were aware of e-cigarettes. For nearly half of the respondents, the major source of information was mass media/internet, followed by friends or acquaintances, while other sources of information were also suggested by 58 (11.6%). 31 (6.2%) participants from our sample reported having used e-cigarettes, which included 6 (1.2%) daily users. By comparison, we found that conventional tobacco users were significantly more likely to have heard of e-cigarettes (87.6% vs 51.6%, p < 0.001) and having used them (13.9% vs 1.3%, p < 0.001). Tobacco non-users were significantly more likely to have heard of e-cigarettes through mass media (46.2%) while users heard through friends/acquaintances (32.9%) (p < 0.03) (Table 1).

**Table 1:** Demographic and details regarding use of e-cigarettes among medical students of Sindh Pakistan (n = 500)

	Total, n (%)	Tobacco users e-cigarettes, n (%)	non-users, n (%)	p-value
No. of students	500 (100)	194 (38.8)	306 (61.2)	
Age in years (mean ± SD)	21.6 ± 1.7	21.7 ± 2.1	21.5 ± 1.5	0.11
Gender				
Male	210 (42.0)	123 (63.5)	87 (28.4)	< 0.001
Female	290 (58.0)	71 (36.5)	219 (71.6)	
Type of institution				
Private	199 (39.8)	77 (39.6)	222 (72.5)	< 0.001
Public	301 (60.2)	117 (60.4)	84 (27.5)	
Year of MBBS				
1st	62 (12.4)	24 (12.4)	38 (12.4)	0.03
2nd	84 (16.8)	26 (13.4)	58 (19.0)	
3rd	94 (18.8)	40 (20.6)	54 (17.6)	
4th	118 (23.6)	58 (29.9)	60 (19.6)	
5th	142 (28.4)	46 (23.7)	96 (31.4)	
Heard about e-cigarettes				
Yes	328 (65.6)	170 (87.6)	158 (51.6)	< 0.001
No	172 (34.4)	24 (12.4)	148 (48.4)	
Source of information^b^				
Friends/acquaintances	103 (20.6)	56 (32.9)	47 (30.1)	0.03
Mass media/internet	123 (24.6)	51 (30.0)	72 (46.2)	
Both	42 (8.4)	31 (18.2)	11 (7.1)	
Other sources	58 (11.6)	32 (18.8)	26 (16.7)	
Use e-cigarettes				
Yes	31 (6.2)	27 (13.9)	4 (1.3)	< 0.001
No	469 (93.8)	167 (86.1)	302 (98.7)	
Frequency of use				
Daily	6 (1.2)	6 (22.2)	0 (0)	
Sometimes	15 (3.0)	14 (51.9)	1 (25.0)	
Rarely (≤ once a month)	10 (2.0)	7 (25.9)	3 (75.0)	

^a^Conventional tobacco product users including cigarettes, shisha and smokeless tobacco. ^b^ Participants who reported knowledge of e-cigarettes

Compared to e-cigarette users majority of non-user perceived that use of e-cigarettes is associated with respiratory diseases like Chronic Obstructive Pulmonary Disease (COPD), Asthma and Lung cancer (45.2% vs 56.6%; p value 0.003), associated with addiction and dependency (29% vs 57.6%; p-value < 0.001) and is harmful for pregnant women (45.2% vs 69.9%; p-value < 0.01) However there was no significant difference of opinion found among users and non-users when they were asked whether e-cigarette help in quitting (p-value 0.38), less harmful than conventional cigarette (p-value 0.20), more harmful than conventional cigarette (p-value 0.28) and equally harmful to conventional cigarette (p-value 0.57). Compared to e-cigarette users, the majority of non-users believe that it is not harmful at all (71.0% vs 62.6%; p value 0.001) (Table 2).

**Table 2:** Knowledge and perception of e-cigarette use among medical students

		e-Cigarette user			Non-user		
		Yes (%)	No (%)	Some what (%)	Yes (%)	No (%)	Some what (%)
1	E-cigarettes can cause respiratory diseases (lung cancer, COPD, asthma)	14 (45.2)	13 (41.9)	4 (12.9)	168 (56.6)	51 (17.2)	78 (26.3)
2	E-cigarettes are associated with addiction/dependency	9 (29.0)	17 (54.8)	5 (16.1)	171 (57.6)	45 (15.2)	81 (27.3)
3	Use of e-cigarettes is harmful in pregnant women	14 (45.2)	12 (38.7)	5 (16.1)	207 (69.9)	31 (10.5)	58 (19.6)
4	E-cigarettes help in smoking cessation	13 (41.9)	13 (41.9)	5 (16.1)	124 (41.9)	95 (32.1)	77 (26.0)
5	Use of e-cigarettes is less harmful than traditional smoking	16 (51.6)	11 (35.5)	4 (12.9)	181 (60.9)	64 (21.5)	52 (17.5)
6	Use of e-cigarettes is more harmful than traditional smoking	4 (12.9)	27 (87.1)	0 (0)	27 (9.1)	250 (84.2)	20 (6.7)
7	Use of e-cigarettes is not harmful at all	10 (32.3)	16 (51.6)	5 (16.1)	29 (9.8)	201 (67.7)	67 (22.6)
8	Use of e-cigarettes is equally harmful as conventional cigarettes	4 (12.9)	22 (71.0)	5 (16.1)	60 (20.2)	186 (62.6)	51 (17.2)

We found that those who were using e-cigarettes were using other tobacco products concomitantly more often than nonuser. Use of conventional cigarette was (80.6%), smokeless tobacco use was (38.7%) and shisha use was (83.9%) among e- cigarette users compared to 13.4, 2.3 and 31.6% of non e-cigarette user respectively. This was also suggested by a multivariable logistic regression analysis demonstrating significantly higher odds of e-cigarette use among those who reported use of conventional cigarettes [OR: 10.6, 95% CI 3.6–30.8, p < 0.001] as well as use of smokeless tobacco products [OR: 7.9, 95% CI 2.7–23.4, p < 0.001], whereas the association was only marginally significant for Shisha use [OR: 3.05, 95% CI 0.9–9.6, p = 0.05] (Table 3).

**Table 3:** Multivariable logistic regression analysis for association of e-cigarette use with other forms of tobacco in medical students

	n (%)	Crude OR (95% CI)	Adjusted OR (95% CI)	p-value*
Conventional cigarettes (ever use)				
Yes	88 (17.6)	26.8 (10.5–68.0)	10.6 (3.6–30.8)	< 0.001
No (ref.)	412 (82.4)	–	–	
Smokeless tobacco use				
Yes	23 (4.6)	26.2 (10.3–67.1)	7.9 (2.7–23.4)	< 0.001
No (ref.)	477 (95.4)	–	–	
Shisha use				
Yes	174 (65.2)	11.2 (4.2–29.9)	3.05 (0.9–9.6)	0.05
No (ref.)	326 (34.8)	–	–	
Gender				
Male	210 (42.0)	4.33 (1.89–9.89)		
Female (ref.)	290 (58.0)	–		
Academic year				
Year I–III	240 (48.0)	1.12 (0.54–2.34)		
Year IV–V	260 (52.0)			
Type of university				
Private	201 (40.2)	1.24 (0.59–2.57		
Public (ref.)	299 (59.8)	–		

* Adjusted model

### Discussion

The popularity of e-cigarettes is currently rising globally while limited data are available on its safety and efficacy, as well as perceptions in young populations who are most susceptible to its uptake. Our study highlights the knowledge and awareness in undergraduate medical students, and is the first study to address this research gap in a developing country. We found that fairly high numbers of respondents were aware of e-cigarette however e-cigarette use was generally low as compared to conventional tobacco products; majority of e-cigarette users were male. These results are similar to study conducted among medical students in Poland [9] however the proportion of ever users of e-cigarette was higher in our study, i.e. 6.2% compared to 3.5%.

A survey of Tobacco use in adults in Montana found 11.2% ever use and 1.3% current use of e-cigarettes [13]; a recent survey from Egypt among the general population found more than half had knowledge about e-cigarette but none of them reported it use [14]. Our study found e-cigarette use was significantly higher among smokers and male gender as reported previously [6, 12]. This is consistent with data on cigarette smoking in Pakistan, which is disproportionately higher in men.

In our study e-cigarettes were mostly perceived as less harmful than conventional cigarettes by both non-users and users. It is alarming that the majority of non-users also thought of e-cigarettes as less harmful. This is possibly likely as it is marketed heavily as a safer alternative, and can potentially lead non-users to start using e-cigarettes in the near future. It is of great concern as noted previously that newer users have often tried it as an experiment especially in teenagers and young populations, and can hence serve as a gateway to nicotine addiction in these groups [6–8].

Only 27.4% of respondents in our study thought e-cigarettes help in quitting, which is low compared to 58.7% of previous study reported in medical students [9]. This may be because e-cigarette is perhaps not widely used and hence not established as a smoking cessation aid in Pakistan. While published data are also conflicting, a recent study from Japan also found negative association between smoking cessation and e-cigarette [15] while a systemic review suggested inconclusive findings due to lack of high quality evidence [11].

It is interesting to note that majority of e-cigarette users thought that it is not harmful during pregnancy and did not claim any association with addiction and dependency. However, data is now emerging on some serious consequences such as eosinophilic pneumonia, lipoid pneumonia and bronchiolitis [16] reported explosion injuries [17] increased heart rate, blood pressure and arrhythmias [18, 19]. It’s benefits are uncertain although it is considered and promoted safer than conventional cigarette but its long term effects are still unknown.

Although e-cigarette are widely advertised as smoking cessation aid but in our study we found majority of e-cigarette users reported dual use of both e-cigarettes and conventional cigarettes. Although only a snapshot, the finding does warrant consideration and was consistent with studies published elsewhere [9, 14]. This pattern of dual use is likely related to high dependency among these participants as seen previously [20, 21]. We have also found strong association of e-cigarette use with conventional cigarette and smokeless tobacco product.

In conclusion, a fairly high number of respondents were aware, although e-cigarette use was not found to be widely popular among the study sample. Users generally considered e-cigarettes to be relatively safer, and demonstrated higher odds of concomitant cigarette smoking and smokeless tobacco use. Considering e-cigarettes less harmful by both users and nonuser is alarming. While further studies from developing countries are required on its safety and efficacy as smoking cessation aid, a periodic surveillance is needed for a better understanding of e-cigarette use in different populations and settings. There should be laws against its promotion and marketing should be prohibited as a safe alternative before having definitive evidence demonstrating its benefits.

## Limitations

This study was done among medical students of developing country Pakistan but it has certain limitations [1]. Our study presents a snapshot of medical students’ use and perceptions, which may differ from other university students enrolled in non-medical universities and colleges; hence limiting generalizability [2]. We also relied largely on self-reported estimates of tobacco and e-cigarette use, misreporting of tobacco and e-cigarette use through self-report cannot be ruled out [3]. We were also unable to gauge the degree of misclassification in self-report as we did not use any biochemical verification techniques due to financial limitations. Further efforts should incorporate a broader study population and use biochemical validation measures.

## Abbreviations

e-cigarette: electronic cigarette
ENDS: electronic nicotine delivery systems
MBBS: Master in bachelor and master in science
CDC: centers for disease control and prevention
COPD: chronic obstructive pulmonary disease
